# Development of a decision analytic model to support decision making and risk communication about thrombolytic treatment

**DOI:** 10.1186/s12911-015-0213-z

**Published:** 2015-11-11

**Authors:** Peter McMeekin, Darren Flynn, Gary A. Ford, Helen Rodgers, Jo Gray, Richard G. Thompson

**Affiliations:** Institute of Health and Society, Newcastle University, Newcastle Upon Tyne, UK; Institute for Ageing and Health (Stroke Research Group), Newcastle University, Newcastle Upon Tyne, UK; School of Health, Community and Education Studies, Northumbria University, Newcastle upon Tyne, UK; Department of Healthcare, Northumbria University, Newcastle Upon Tyne, UK

**Keywords:** Acute Cerebral Infarction, Emergency treatment of Stroke, Thrombolysis, Clinical Decision Support, Predictive Models

## Abstract

**Background:**

Individualised prediction of outcomes can support clinical and shared decision making. This paper describes the building of such a model to predict outcomes with and without intravenous thrombolysis treatment following ischaemic stroke.

**Methods:**

A decision analytic model (DAM) was constructed to establish the likely balance of benefits and risks of treating acute ischaemic stroke with thrombolysis. Probability of independence, (modified Rankin score mRS ≤ 2), dependence (mRS 3 to 5) and death at three months post-stroke was based on a calibrated version of the Stroke-Thrombolytic Predictive Instrument using data from routinely treated stroke patients in the Safe Implementation of Treatments in Stroke (SITS-UK) registry. Predictions in untreated patients were validated using data from the Virtual International Stroke Trials Archive (VISTA). The probability of symptomatic intracerebral haemorrhage in treated patients was incorporated using a scoring model from Safe Implementation of Thrombolysis in Stroke-Monitoring Study (SITS-MOST) data.

**Results:**

The model predicts probabilities of haemorrhage, death, independence and dependence at 3-months, with and without thrombolysis, as a function of 13 patient characteristics. Calibration (and inclusion of additional predictors) of the Stroke-Thrombolytic Predictive Instrument (S-TPI) addressed issues of under and over prediction. Validation with VISTA data confirmed that assumptions about treatment effect were just. The C-statistics for independence and death in treated patients in the DAM were 0.793 and 0.771 respectively, and 0.776 for independence in untreated patients from VISTA.

**Conclusions:**

We have produced a DAM that provides an estimation of the likely benefits and risks of thrombolysis for individual patients, which has subsequently been embedded in a computerised decision aid to support better decision-making and informed consent.

## Background

The risks and benefits of thrombolysis for acute ischaemic stroke vary from patient to patient depending on their clinical characteristics. Even within licensing criteria, clinicians have expressed a desire for individualised predictions [1]. Although predictive models for thrombolytic treatment exist [2–4] the majority are derived from single or pooled analyses of randomised controlled trials (RCTs) [5–9]. Rothwell [10] has highlighted the issue facing doctors of assessing external validity when taking evidence from RCTs into account in determining whether their results can be reasonably applied to patients treated in routine practice. RCTs are infrequently powered to identify all factors associated with the range of outcomes following treatment. For example, the rate of symptomatic intracerebral haemorrhage (SICH) is reported in trials, but the low rates make identification of the factors associated with this outcome difficult, yet the risk of haemorrhage is a factor in the decision to offer thrombolysis. Doctors have to rely on information from observational studies where factors associated with SICH have been identified. Decision analytic models (DAMs) are an established and explicit way to synthesise available evidence about the outcomes of healthcare interventions [11]. This paper describes the development of a DAM that brings together results from RCTs together with observational data to support decision making about thrombolysis for individual patients. Developing a DAM of this type means identifying, combining and validating the best sources of evidence and data about the outcomes of interest in as methodologically a robust way as possible. The DAM’s uses include the communication of likely risks, benefits and prognosis to patients/relatives during the hyper-acute stroke period, supporting informed consent, promoting patient/family involvement in decision-making and modelling the implications of stroke-related service developments.

In a previous paper we reported improved explanatory power and relevance of a predictive model derived from large scale RCTs of thrombolysis, the Stroke-Thrombolytic Predictive Instrument (S-TPI) [12], with respect to outcomes in individual patients seen in clinical practice, by calibration with patient data from routine practice [13]. Calibration is the process whereby predicted outcomes are compared to observed outcomes. In the S-TPI the probability of a ‘good outcome’ is dependent on age, systolic blood pressure (SBP), diabetes, sex, stroke severity (National Institutes of Health Stroke Scale (NIHSS)), previous stroke, onset to treatment time and thrombolysis. Its predictions are made in terms of the modified Rankin score (mRS) [14]. The mRS measures the degree of disability in carrying out daily activities on a six point scale and a seventh point denoting death. Age, stroke severity and serum glucose are predictors of a ‘catastrophic outcome’ an mRS of 5 or 6. The S-TPI defined a ‘good outcome’ as a mRS of ≤ 1 i.e.”*able to carry out all usual activities, despite some symptoms*”. This definition is discordant with the definition more typically used in clinical practice where mRS ≤ 2 i.e. “*able to look after own affairs without assistance, but unable to carry out all previous activities*” characterises a ‘good outcome’. The S-TPI does not include explicit predictions for SICH and related outcomes (subsequent independence, dependence and death), which are also consequences of the decision to treat or not to treat although the predictions are implicit in the S-TPI’s three-month outcomes. We therefore aimed to develop a DAM by re-calibrating the original S-TPI using data about patients treated in routine practice, as well as incorporating risk of SICH and related outcomes.

## Methods

### Overview

The DAM was created using the predictions of the S-TPI calibrated with SITS data. This enhanced model allows prediction, for any set of inputs representing a patient, of the probability of being in any one of the three states: death (mRS = 6), dependence (2 ≤ mRS < 6) and independence (mRS ≤ 2). Separately we calculated the risk of SICH for that patient, if treated, and from what we know about post-SICH outcomes we estimated the probability of: SICH leading to death, dependence and independence. These SICH outcomes were combined with the calibrated S-TPI into the DAM.

### Calibration of the predictive model of independence (mRS ≤ 2) death and dependence

Calibration curves were constructed to establish the accuracy of the S-TPI predictive equations for mRS ≤ 2 and mRS = 6 in treated patients using data from SITS-UK [14] and to confirm calibration was necessary. The SITS-UK dataset contains information about patients who were treated intravenous thrombolysis and includes outcome data in the form of the mRS as well as the predictors used in the DAM [12–14]. Calibration curves show whether predictions from the S-TPI correspond with outcomes in the SITS-UK population and how any under or over prediction varies with outcome probabilities.

We applied the same data analysis strategy used to calibrate the S-TPI in our previous report [12], adjusting the S-TPI model because no association between thrombolytic treatment and a catastrophic outcome (severe disability or death, mRS 5 to 6) at three months exists and SITS-UK patients might reasonably be expected to have a different mortality risk than the patients in the RCTs. Because death before three months is a competing risk to a normal outcome, only cases surviving (mRS ≤ 5) at three months were used in the calibration of S-TPI to predict mRS ≤ 2. We also assumed that probability of death associated with risk of SICH would be captured in the overall probability of death in treated patients. Further analyses were undertaken to establish the improvement in explanatory power of the model by including predictors of independence (e.g. signs of current infarction on pre-treatment brain scan, congestive heart failure, and blood glucose) identified from the research literature [14] and our previous work [13]. The record of signs of new current infarction were taken from Safe Implementation of Thrombolysis in Stroke-Monitoring Study (SITS-MOST), defined as “Baseline CT examinations evaluated for early infarct signs (hypodensity, dense artery sign)” [15] as recorded by the responsible physician. SITS-MOST was an observational study that assessed the safety profile of Alteplase, the drug used in thrombolysis [16].

*The method of calibration consisted of logistic regressions with the observed outcome (death or independence from SITS-UK data) as the dependent variable. Alongside the independent variables used in the construction of the S-TPI an additional independent variable was included. This additional variable was the predicted probability of the outcome of interest derived from the S-TPI. In the case of ‘independence’ additional independent variables were tested in the regression where evidence suggested their potential in improving the explanatory power of the model. As with the identification of independent variables used in the S-TPI, a stepwise approach was used to delete from the model, in turn, the independent variable whose removal most improved the model to the point that removal of further variables no longer improve the model.* Consequently, any statistical significance of remaining coefficients implies association with under or over prediction. Coefficients associated with either under or over prediction were applied to both the treated and untreated predictions in order to maintain the (net) treatment effect of thrombolysis (absolute difference in probability of independence [mRS ≤ 2] in treated and untreated patients). If prediction discrepancies were found to be associated with independent variables interacting with treatment effect we assumed that the treatment effect reported by the S-TPI was correct, and applied the calibration coefficients to both the treated and untreated outcome predictions. To validate our assumptions about predictions of outcomes in patients who do not receive thrombolytic treatment, we compared outcomes in untreated patients (*N* = 4,360) recorded in Virtual International Stroke Trials Archive (VISTA) [17] and compared the C-statistics, the generalised form of the area under the receiver operating curve (AUC) [18], of the S-TPI to the calibrated S-TPI. VISTA is a database containing anonymous data about individual patients from completed clinical trials of treatments for stroke. C-statistics, represent the probability that the prediction is better than chance. They range from 0.5 to 1.0, with 0.5 representing a model no better than chance and 1.0 a model that perfectly predicts.0.7 is typically considered reasonable and 0.8 strong [19].

A stepwise logistic regression analysis was performed using SITS-UK (*N* = 2,401) data to establish statistically significant predictors of death in the S-TPI at three months in treated patients. We also investigated whether the addition of blood glucose and the presence of an infarct on brain scan enhanced the prediction qualities of the model for death. Dependence (mRS 3 to 5) at three months was calculated as unity minus the sum of the probabilities for independence and death.

To quantify the improvement in the predictive abilities of the S-TPI, we tested its predictions for mRS ≤ 2 using the SITS-UK dataset. Receiver operating curves (ROC) were used to estimate the ability of the S-TPI at three months to discriminate (i) between treated patients who do and don’t benefit (mRS ≤ 2) from thrombolysis, and (ii) between treated patients who die (mRS = 6) or survive (mRS ≤5). C-statistics were used to compare the ability of the S-TPI to discriminate between patients that would benefit or not from thrombolytic treatment.

### Prediction of SICH and related outcomes

We used the SITS-MOST definition of SICH ‘*NIHSS scores worsening ≥ 4 within 24 h and an intracerebral haemorrhage type PH2 (a space occupying hematoma of >30 % of the infarct zone with substantial mass effect attributable to the hematoma)*’ [20]. Cases in the SITS-UK data that met the criteria for the SITS-MOST definition of SICH were too few (*n* = 18) to derive a prediction equation; therefore the risk of SICH for treated patients was estimated used a scoring model reported in the literature [21] derived from the wider SITS-MOST population. A suitable predictive equation for outcomes following SICH could not be identified. We used the following proportions that mapped onto mRS ranges in our DAM: 6 % (mRS ≤ 2), 33 % (mRS 3 to 5) and 61 % (mRS 6) [20].

### Data

Information about 4022 patients who were thrombolysed between December 2002 and February 2010 were obtained from SITS-UK. Cases with incomplete or unconfirmed data for mRS at three months were excluded (*n* = 227). We also applied range restrictions to predictors of mRS at three months in the S-TPI (age ≥18, glucose ≤ 25 mmol; systolic blood pressure ≤ 200 mm Hg; and onset time to treatment ≤ 270 min), yielding sample sizes of 1,996 for analysis of mRS 0 to 2 and 2,401 for analysis of death at three months (Table 1). The table also describes the VISTA untreated population used to validate outcomes in untreated patients. VISTA collates and provides access to completed, anonymised RCT data for the purposes of novel exploratory analyses. The data used to estimate the risk of SICH was from patients in the international data set SITS-MOST; details of these patients are reported elsewhere [21].

**Table 1:** Baseline characteristics of S-TPI cases and those in SITS-UK and VISTA with mRS of ≤ 2 pre treatment

	S-TPI *N* = 2,131		SITS-UK, *N* = 2,401		VISTA untreated *N* = 4,630
Characteristic		Cases omitted from analyses (*n* = 227)	Patients: surviving at 3-months (*n* = 1,996)	Patients: not surviving to 3-months (*n* = 405)	
Age mean (SD)	65.9 (11.4)	67.8 (13.26)	66.7 (12.7)	73.3 (11.3)	70.5 (12.2)
Sex, % male	54.7 %	58.6 %	58.7 %	56.5 %	51.6 %
NIHSS score (median, IQR)	12 (8,17)	12 (7,18)^b^	12 (8,17)	19 (15,22)	13 (8,18)
Hypertension %	58.8 %	60.36 %	58.0 %	62.5 %	72.1 %^a^
Diabetes %	20.8 %	14.9 %	12.0 %	17.3 %	21.4 %
Prior stroke %	16.6 %	14.41 %	13.6 %	14.8 %	34.3 %^a^
Atrial fibrillation %	18.6 %	27.0 %	23.6 %	30.6 %	31.6 %
Onset time to treatment (OTT) minutes (median, IQR)	235 (155,290)	146 (109,175)	150 (120, 175)	150 (120, 178)	-
OTT, % within 3–4.5 h	61.3 %	15.3 %	16.6 %	17.3 %	-
Systolic blood pressure, mm Hg (mean, SD)	152.6 (20.3)	145.5 (21.8)^a^	146.9 (20.8)	147.9 (21.0)	156.8 (26.7)
Serum glucose mmol/l (median, IQR)	6.78 (5.83,8.58)	6.2 (5.6,7.8)	6.2 (5.4, 7.5)	6.9 (6.0, 8.4)	6.7 (5.8,8.5)
Signs of current infarction on pre-treatment scan %	NA	23.9 %	24.3 %	34.8 %	NA
Congestive heart failure %	12.1 %	4.5 %	4.6 %	6.9 %	NA

^a^ignoring missing values, ^b^National Institutes of Health Stroke Scale

S-TPI = Stroke-Thrombolytic Predictive Instrument

SITS-UK = Safe Implementation of Treatments in Stroke UK

## Results

The calibration curves showed that the S-TPI under- and over-estimated the probability of mRS ≤ 2 and mRS 6 in SITS-UK patients respectively, confirming that calibration was warranted. The C-Statistics were 0.785 for a good outcome (mRS ≤ 2) and 0.770 for death.

### The calibrated S-TPI Model for independence (mRS ≤ 2) in treated patients

We found the original S-TPI accounted for 91 % of the variability in ‘predicted’ probability of independence in patients that survived for three months. Full details can be found in Table 2. In the S-TPI, treatment interacts with sex, onset time to treatment (OTT), systolic blood pressure (SBP), stroke severity and previous stroke. Calibration determined that sex, diabetes, prior stroke and onset time to treatment were not statistically significant predictors of discrepancies between the original S-TPI’s predictions of independence and actual independence in the SITS-UK population. Age, NIHSS score and systolic blood pressure were statistically significant in the calibration of the S-TPI. The impact of increasing systolic blood pressure on reducing the probability of a good outcome in the S-TPI was lessened as was the effect of being male. Age and stroke severity reduced the probability of a good outcome more than predicted by the S-TPI. However for stokes with an NIHSS score of 17 and over the probability of a good outcome rose as age increased. In addition signs of current infarction on pre-treatment imaging were associated with improved prediction.

**Table 2:** Results of calibration of S-TPI on mRS ≤ 2 and on death

Parameter	S-TPI (mRs ≤1)	DAM correction factor (mRs ≤ 2)	S-TPI (mRs > 4)	DAM correction factor (mRS > 5)
Intercept	1.0702	−0.1144	−7.580	−7.417^a^
Thrombolysis Treatment	3.3774			
Age (per 1 year increase)	0.0173	−0.0259^c^	0.050	0.0418^a^
Systolic blood pressure, SBP (per 1 mmHg)	−0.00488	0.00831^c^		
Diabetes	0.7431			
Male (vs. Female)	0.3757	0.1763^d^		
NIHSS^e^ (per 1 unit increase)	−0.00764	−0.1372^b^	0.142	0.132^a^
Prior stroke	0.3728			
Onset to treatment, OTT (per 1 min increase)	0.000333			
Treatment^*^ SBP	−0.0117			
Treatment^*^ Male	−0.4286			
Treatment^*^ Prior stroke	−0.7738			
Treatment^*^ OTT	-			
Age^*^ NIHSS	−0.00285	0.00159^c^		
Prediction of S-TPI	NA	3.3896^a^	NA	0
Presence of infarct on brain scan	NA	−0.4020^a^		
Serum glucose (mmol/L, truncated at 25)	-	-	0.072	0.1024^a^

Signif. codes: >0.001^a^; 0.001^b^; 0.01^c^; 0.05^d^

^e^National Institutes of Health Stroke Scale

### The calibrated S-TPI model for prediction of death

The S-TPI’s predictions for a catastrophic outcome (mRS 5 to 6) and the calibration for death (mRS 6) derived from SITS-UK are given in Table 2. All parameters were statistically significant predictors of death, which was consistent with the S-TPI model for catastrophic outcomes, although glucose in the DAM has a larger negative effect on probability of death than in the S-TPI.

### Properties of the calibrated models for independence and death, and validation of untreated outcomes using VISTA data

**Fig. 1:**
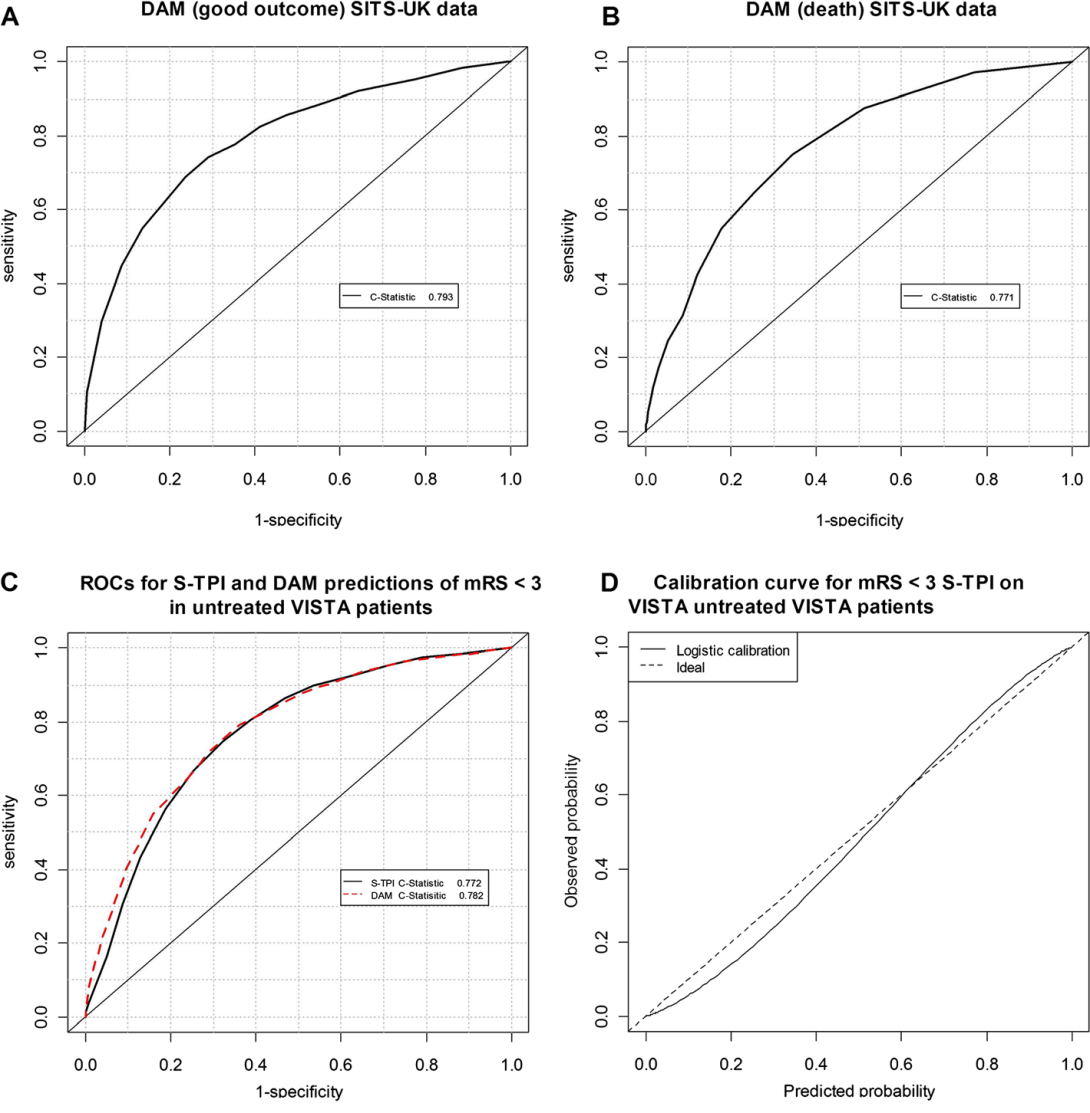
ROC curves for the calibrated S-TPI for treated (A&B) and untreated patients (C&D)

The calibrated S-TPI models for independence (mRS ≤ 2) showed an increase in the-C-statistic from 0.785 to 0.793 (Fig. 1a). The C-statistic when predicting death was 0.771 in the calibrated S-TPI compared to 0.770 in the original (Fig. 1b). The ROC curves for predictions of independence in VISTA patients using the original and calibrated S-TPI models are shown in Fig. 1c. Compared with the original S-TPI model for independence in untreated patients, the calibrated S-TPI has improved discrimination in predicting independence in untreated patients from VISTA; it under- and over predicts at lower and higher probabilities of independence respectively (Fig. 1d).

**Fig. 2:**
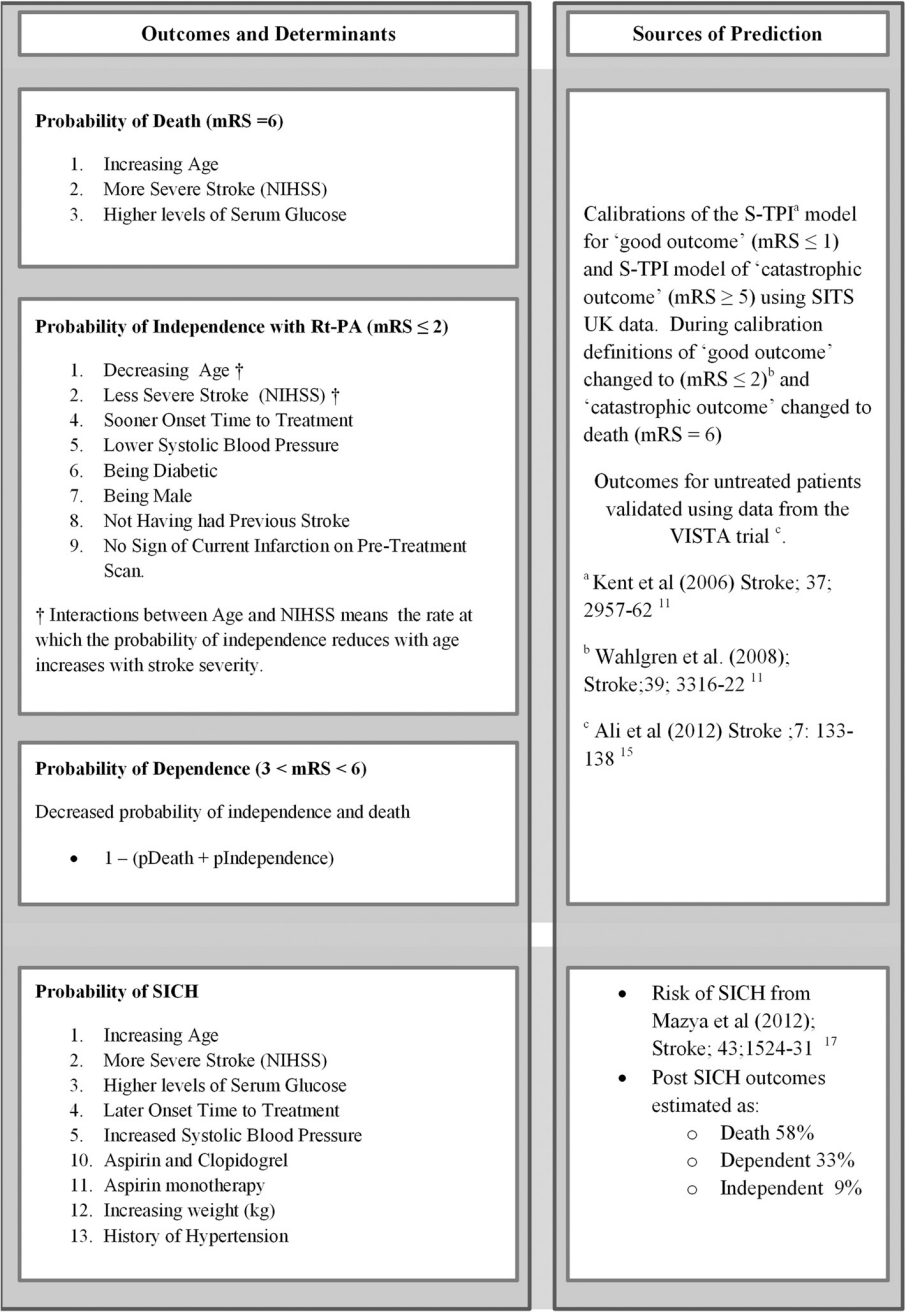
Decision Analytic Model, its inputs and predictions

An overview of the DAM showing its inputs and their relationships is show in Fig. 2.

### Predictions of the decision analytic model

In their paper describing the development of the S-TPI, Kent and colleagues [11] presented the predicted outcomes of a group of patients with and without thrombolysis. In Table 3 we report these outcomes alongside the predictions from our calibrated version of the S-TPI. Missing values were imputed using appropriate values from the SITS-UK database. Reflecting the modified definition of a good outcome (mRS ≤ 2instead of mRS ≤ 1), the DAM predicts a greater likelihood of a good outcome in untreated patients. With thrombolysis the probabilities were in the main greater than these predicted by the S-TPI. Predictions of a catastrophic outcome/ death are lower in the DAM, as mRS = 5 is no longer included in this category. The final column shows the probability of SICH in treated patients regardless of final outcome which is captured in the mRS ≤ 2 mRS > 5 values.

**Table 3:** Individual patient predictions

Patient characteristics								S-TPI			DAM			
Age	Gender	Diabetes	Prior Stroke	SBP (mm Hg)	Glucose (mmol/L)	NIHSS	OTT	mRS ≤ 1		mRS ≥ 5	mRS ≤ 2		mRS > 5	SICH
								No rtPA	rtPA		No rtPA	rtPA		
77	F	Yes	No	140	15.2	5	179	48 %	72 %	13 %	54 %	69 %	12 %	3.14 %
57	M	Yes	No	179	20.7	5	164	51 %	56 %	7 %	73 %	76 %	9 %	3.14 %
73	F	No	No	160	7.1	10	113	36 %	63 %	12 %	51 %	69 %	9 %	3.14 %
76	F	Yes	Yes	140	15.7	12	170	21 %	27 %	28 %	21 %	24 %	26 %	3.72 %
73	F	No	No	170	6.4	16	89	13 %	30 %	24 %	29 %	41 %	17 %	3.14 %
64	M	No	No	169	7.4	18	175	16 %	21 %	22 %	34 %	37 %	17 %	3.14 %
75	M	No	No	169	7.2	19	165	10 %	13 %	35 %	26 %	28 %	26 %	3.72 %
77	M	No	No	150	4.7	19	90	10 %	20 %	33 %	25 %	31 %	23 %	3.14 %
51	F	No	No	165	13.1	29	122	4 %	9 %	51 %	8 %	10 %	47 %	5.05 %

Imputing SITS-UK mean infarct = 0.294; Weight 80 kg; Aspirin Yes; Clopidogrel No; Hypertensive No

### One-way sensitivity analyses

**Fig. 3:**
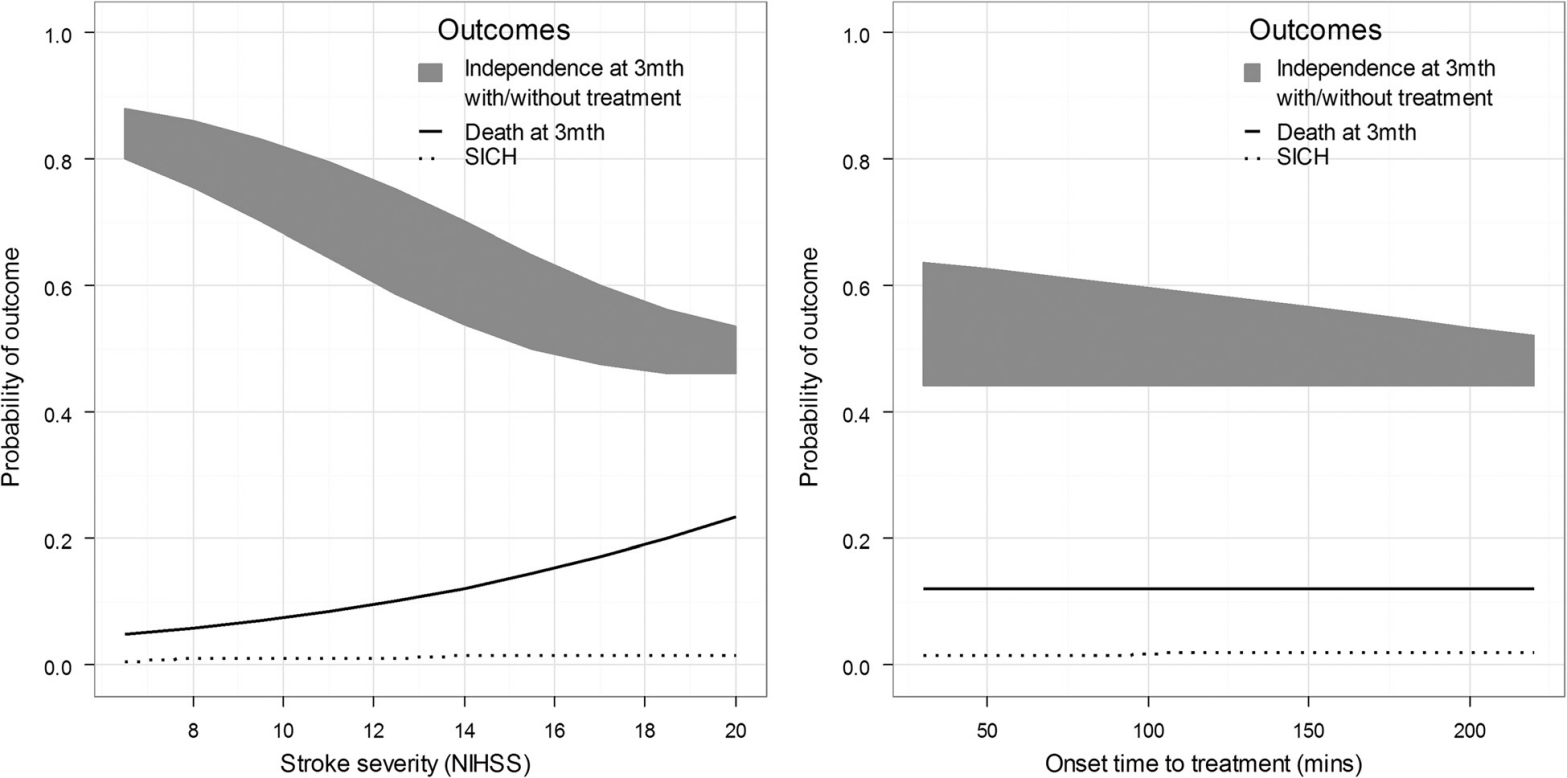
Individual predictions of the S-TPI and DAM

The DAM’s predictions across a range of values of one input, whilst holding others constant, are shown in Fig. 3. Other characteristics used in the predictions are a 70 year old male, who is not diabetic, has not previously suffered a stroke, systolic BP 140 mm/Hg, blood glucose 6.5 mmol/l, and scored 14 on the NIHSS scale, was treated in 90 min and had no infarction present on pre-treatment scan. In each plot the broken lines show the probability of SICH increases slightly with stroke severity and time to treatment. The probability of death, shown by the solid lines, also increases with stroke severity but is unaffected by time to treatment. The grey areas represent the potential gain in probability of independence.

## Discussion

We have addressed the external validity issues of the S-TPI by incorporating the outcomes of patients treated in routine clinical practice. By calibrating the S-TPI to accommodate the outcomes identified by clinicians as most relevant to routine practice, adjusting its structure to isolate the effect of death, and incorporating predictors of additional outcomes of interest, we have developed a tool that captures the variation in outcomes associated with individual patient characteristics. We were able to validate our assumptions about outcomes in untreated patients using a second data set and concluded our assumptions were valid. We have also addressed the feature of the S-TPI where there are no explicit predictions of adverse effects of thrombolysis as raised by Whitley et al. [22] and Emberson et al. [23]. This is a result of the (valid at a population rather than the individual level) assumption that the net effect of thrombolysis on death is zero because any increases in deaths in the acute phase of stroke caused by thrombolysis are offset by lives saved in the post-acute phase. Our prediction of SICH quantifies the risks associated with thrombolysis. As with any treatment decision, the key issue is the balance between risks and benefits. The threshold of SICH risk at which a physician might choose not to treat, or a patient elects not to receive treatment, depends on the potential benefits of treatment. The predictions allow physicians to weigh up the risks and benefits of treating any individual patient. For example, the 75 year old male patient (in Table 3) treated at 165 min with a NIHSS of 19 has a 26 % chance of being independent without thrombolysis and a 28 % chance when thrombolysed, yet has a 3.7 % of SICH if treated. This prediction, in part, addresses the criticisms of Whitley et al. [22] and Emberson et al. [23] that predictions made by tools like the S-TPI and our DAM always predict benefit, they do identify patients where that benefit is very small.

However, there are potential weaknesses of our approach. The SITS-UK data used to calibrate the S-TPI was itself informed by the results of the original RCTs, and patients less likely to have good outcomes following thrombolysis are less likely to appear in SITS-UK. The finding that the probability of a good outcome for stroke patients with an NIHSS score of 17 and over rose as age increased indicates potential selection bias. New research into the risks of haemorrhage amongst untreated patients would allow the DAM to reduce the potential over prediction of risk of haemorrhage in treated patients*.* There is a risk of over prediction of haemorrhage in treated patients because we have not included the risk of haemorrhage in untreated patients. This is likely to be small, and we decided not to include the prediction of SICH in untreated patients because of the limited availability of data. The third International Stroke Trial (IST3) [24] reported 6.5 times the number of SICH in treated patients compared with patients not thrombolysed at seven days when treatment was given within three hours.

**Fig. 4:**
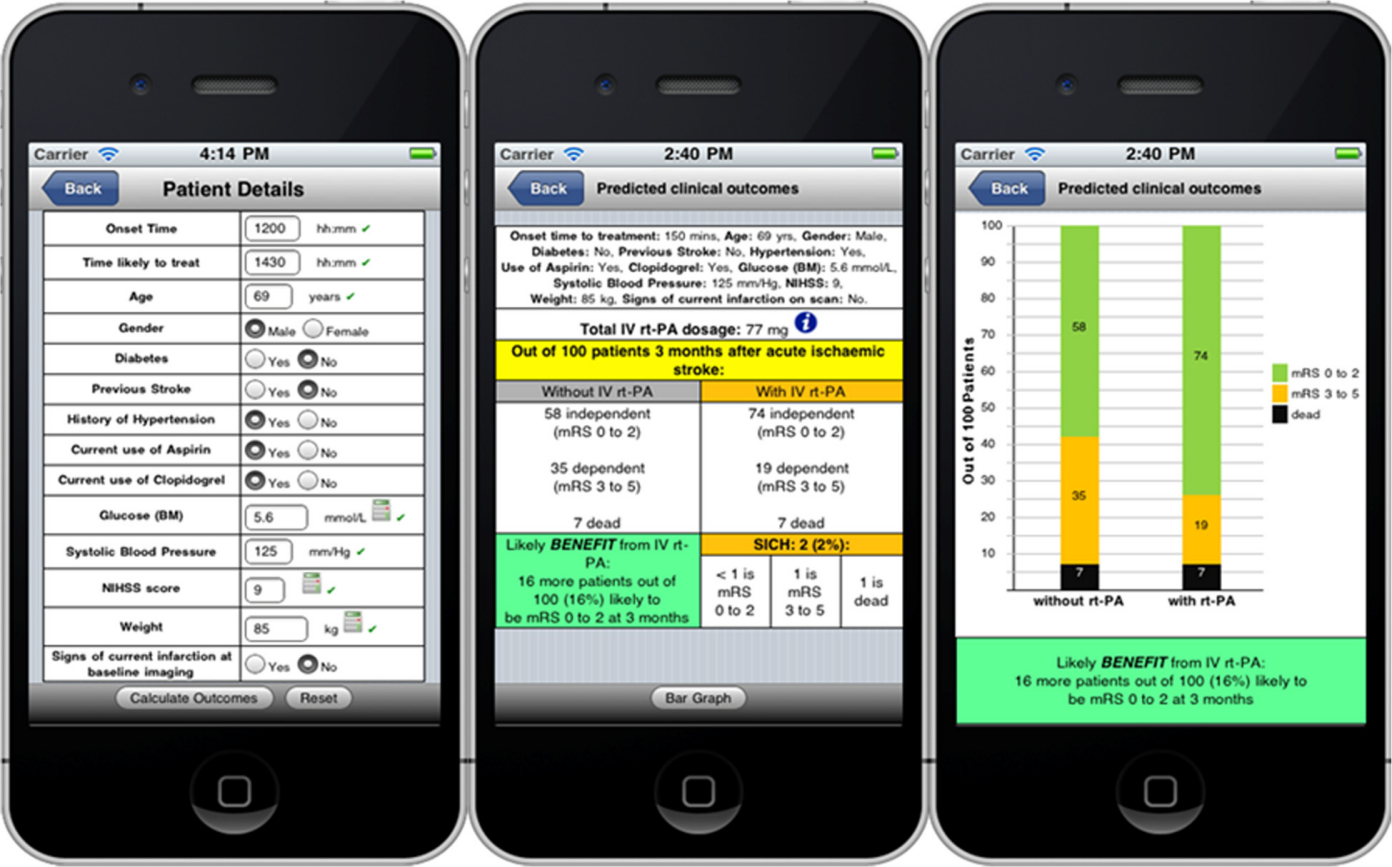
Prototype version of COMPASS

The decision to offer any treatment involves consideration of the probability and magnitude of benefits and the risk and severity of any harms. Unlike the S-TPI, we included explicit risks of SICH resulting from thrombolysis alongside the estimates of likely benefit to highlight this trade-off. The subsequent structured development process by which the DAM was embedded into a computerised decision aid for stroke thrombolysis (COMPASS), including a mixed methods feasibility testing of a resultant gamma prototype in clinical practice is described in a sister paper (Flynn et al. [25]). Briefly, COMPASS was used in a pragmatic fashion by 10 stroke clinicians in three acute stroke units for patients eligible for thrombolysis. Findings demonstrated usability and acceptability of COMPASS amongst patients, relatives and clinicians to support clinicial decision making or to obtain more detail on likely patient benefit after a decision to offer thrombolysis. (in particular for patients at the extremes of the licensing criteria; for example low NIHSS scores) and interpretation of risks and benefits of thrombolysis, including overall net benefit for individual patients were facilitated by the use of graphical risk presentations, specifically pictographs showing outcomes with and without thrombolysis at 3 months. The potential of COMPASS as a clinical training aid was also emphasised for clinicians, as well as an adjunct to the telemedicine model of stroke care was also emphasised by clinicians. The results of this research are presented in a sister paper [25] and an example of a prototype application embedding the DAM is shown in Fig. 4.

## Conclusion

A pragmatic approach to developing a model to provide individualised outcome prediction for thrombolysis based on individual patient characteristics has resulted in a model that reflects the needs of clinicians. This was achieved by incorporating feedback from clinicians about what outcomes are important to support better decision making with evidence about outcomes of patients treated in routine practice, alongside the best evidence on effectiveness from RCTs. This predictive decision analytic model differs from previous models as it combines evidence from a range of sources, including trials and observational studies, to support decision making. Building on the individualised predictions of the S-TPI, our decision analytic model has enhanced external validity and improved clinical applicability to likely outcomes for individual patients.

## Abbreviations

COMPASS: COMPuterised decision Aid for Stroke thrombolysis
DAM: Decision Analytic Model
IST3: The third International Stroke Trial
NIHSS: National Institutes of Health Stroke Scale
mRS: modified Rankin Score
RCT: Randomised Controlled Trials
ROC: Receiver Operating Curves
SBP: Systolic Blood Pressure
SICH: Symptomatic IntraCerebral Haemorrhage
S-TPI: Stroke-Thrombolytic Predictive Instrument
SITS-MOST: Safe Implementation of Thrombolysis in Stroke-Monitoring Study
SITS-UK: Safe Implementation of Treatments in Stroke - UK
VISTA: Virtual International Stroke Trials Archive
