# Erratum to: Development of a decision analytic model to support decision making and risk communication about thrombolytic treatment

**DOI:** 10.1186/s12911-016-0242-2

**Published:** 2016-01-16

**Authors:** Peter McMeekin, Darren Flynn, Gary A. Ford, Helen Rodgers, Jo Gray, Richard G. Thomson

**Affiliations:** 1 Institute of Health and Society, Newcastle University, Newcastle Upon Tyne, UK; 2 Institute for Ageing and Health (Stroke Research Group), Newcastle University, Newcastle Upon Tyne, UK; 3 School of Health, Community and Education Studies, Northumbria University, Newcastle upon Tyne, UK; 4 Department of Healthcare, Northumbria University, Newcastle Upon Tyne, UK

The original version of this article [1] contained an error. After publication it came to the authors’ attention that one of the authors’ names was incorrectly spelt. The original spelling reads as ‘Richard G. Thompson’, however the correct spelling should be Richard G. Thomson.

## References

[1] McMeekin P (2015). Development of a decision analytic model to support decision making and risk communication about thrombolytic treatment. Cancer Cell Int.

